# Localization algorithms for asynchronous time difference of arrival positioning systems

**DOI:** 10.1186/s13638-017-0851-1

**Published:** 2017-04-11

**Authors:** Shuai He, Xiaodai Dong, Wu-Sheng Lu

**Affiliations:** Department of Electrical and Computer Engineering, University of Victoria, 3800 Finnerty Road, Victoria, V8P 5C2 Canada

**Keywords:** Localization, Asynchronous positioning systems, Time difference of arrival (TDOA), Semidefinite programming (SDP), Taylor series, Constrained least-squares, Cramer-Rao lower bound (CRLB)

## Abstract

An asynchronous time difference of arrival (ATDOA) positioning system requires no time synchronization among all the anchor and target nodes, which makes it highly practical and can be easily deployed. This paper first presents an ATDOA localization model, and then primarily focuses on two new localization algorithms for the system. The first algorithm is a two-step positioning algorithm that combines semidefinite programming (SDP) with a Taylor series method to achieve global convergence as well as superior estimation accuracy, and the second algorithm is a constrained least-squares method that has the advantage of low complexity and fast convergence while maintaining good performance. In addition, a novel receiver re-selection method is presented to significantly improve estimation accuracy. In this paper, we also derive the Cramer-Rao lower bound (CRLB) of the ATDOA positioning system using a distance-dependent noise variance model, which describes a realistic indoor propagation channel.

## Introduction

Position information brings enormous benefits to many real-life applications ranging from cargo tracking, tourist guiding, emergency evacuation, to countless usage scenarios. As mobile devices become ubiquitous, contextual awareness applications have become popular, and the indoor positioning system has gained significant attention. The time-based localization method, including one-way time of arrival (TOA) and time difference of arrival (TDOA), exploits the fine delay resolution property of wideband signals and has great potential for providing high accuracy location estimation. However, both methods face a major challenge, that is, synchronization is required among the clocks of the involved nodes with a timing accuracy proportional to the desired localization precision.

Efforts have been made in the literature to relax the synchronization requirements, and two common methods are two-way ranging [1, 2] and elliptical localization [3–5]. In two-way ranging, an anchor node transmits a packet to a target node, which replies by an acknowledgment packet to the anchor node after a response delay. The two-way ranging eliminates the error due to imperfect synchronization between nodes, yet this approach is sensitive to clock non-idealities [3, 6]. The elliptical localization system starts with an anchor transmitter (Tx) emitting a pulse, and upon arrival, the pulse is re-transmitted by the target node. An anchor receiver (Rx) captures two pulses in a row, one from the anchor Tx and the other from the target. The time difference between the two received signals can be measured, and together with the knowledge of the anchor Tx and Rx positions, the sum of the distances between the target and the two anchor nodes can be calculated. Hence, the target node lies on the trajectory of an ellipse with anchor Tx and anchor Rx as the two foci. Several elliptical localization systems have been studied in the literature [3–5]. These systems work in a similar manner, and they differ in one or two respects. The system deployment in [3] has a designated anchor Tx emitting an ultra-wideband pulse, and three anchor Rx nodes to perform the time difference arrival measurements. Wang et al. [4] proposed an asymmetric trip ranging protocol, and the system deployment is similar to [3], but it involves a timing logic at the target node, which suffers from clock non-idealities. In [5], a distributed localization scheme is proposed, and it uses the target node to measure the TDOA. Due to the cost and power constraints on the target node, low-performance clocks are normally employed which limits the accuracy. In this paper, we first present a new elliptical localization system, namely, an asynchronous time difference of arrival (ATDOA) positioning system. The ATDOA system’s deployment is different from [3] in that there is no need for a designated anchor Tx. Rather, the proposed simplest deployment contains one anchor Rx and three anchor Tx. A more comprehensive setup contains four transceiver anchors, each of which can be dynamically configured into a Tx or Rx in order to minimize estimation error by performing novel receiver re-selection. More importantly, two new location estimation algorithms tailored for the ATDOA system are proposed and studied. More details can be found at [7].

Due to the imperfect implementation of location sensing systems, lack of bandwidth, added thermal noise, multipath of the radio propagation channel, and the drift of the clocks, there are always errors associated with measurements of location related metrics. To obtain an estimate of target location in the presence of measurement errors, a variety of direct and iterative positioning algorithms have been developed. When measurement error distribution is available, a maximum likelihood estimator (MLE) is commonly used. An approximate maximum likelihood (ML) algorithm was developed in [8] to achieve near-optimal performance without the complexity of “full” maximum likelihood estimation. In [9], a ML-based algorithm was proposed, and simulation results reveal that the solution closely approaches the fundamental bounds. In spite of attaining optimum estimation performance, the ML approach requires sufficiently precise initial estimates for global convergence. In [10], it has been shown that the positioning accuracy of the ML methodology attains Cramer-Rao lower bound (CRLB) at sufficiently small noise conditions. However, it is difficult to implement in practice because the ML cost function contains multiple local minima and maxima, hence, its maximization is sensitive to initial conditions, and there is no guarantee of global optimality [11]. In [12], results show that even when the ML estimator is initialized by a weighted least squares estimate, which is close to the global solution, it still converges occasionally to a local minimum. Unlike the ML approach, the least squares (LS) approach does not assume any characterization of the noise statistic affecting the observations; hence, it is deemed a suboptimal method [13]. However, it has low computational complexity and therefore is easy to implement in a practical system. Basically, there are two approaches for solving the non-linear LS equations. The first approach is to solve them directly in a non-linear least squares (NLS) or weighted least squares (WLS) framework [14–16]. The common procedure is linearization followed by gradient searches. Although optimum estimation performance can be attained, it requires sufficiently precise initial estimates for global convergence because the corresponding cost functions are multimodal. The second approach is linear least squares (LLS) method. It reorganizes the non-linear equations into a set of linear equations so that real-time implementation is allowed, and global convergence is ensured [11, 17–21].

Although the MLE has the highest accuracy, it is highly non-linear and does not assure global convergence. It is possible to relax the ML formulation to a semidefinite programming (SDP) problem in order to provide a high-fidelity approximate solution that can be obtained in a globally optimum fashion with reduced computational efforts. Hence, we first develop a two-step algorithm that takes advantage of the SDP’s global convergence property to provide a solution, that is then used as an initial estimate for a Taylor series method to achieve superior accuracy. The two-step method provides accurate solutions at a cost of considerable computational complexity, and it may not be an ideal approach for applications where computational resources are limited. Therefore, we also present a constrained least-squares (CLS) estimator that provides good solution accuracy with reduced complexity for ATDOA positioning systems.

In addition to a new system deployment, our paper is different from the previous elliptical localization papers [3–5] in several other ways:

- A practical CRLB has been derived for the A-TDOA system. We model the received signal’s signal-to-noise ratio (SNR) as a distance-dependent parameter to derive a more accurate and a more practical lower bound.
- A two-step (SDP + Taylor) algorithm and a CLS algorithm are proposed to estimate the target position in the ATDOA system. The two-step estimator can be applied in applications where accuracy is the most critical, and the CLS estimator is very useful in real-time systems and mobile devices where battery life and computational capability is limited.
- The localization algorithm’s performance is thoroughly studied based on practical achievable ranging accuracy. This unique analysis method allows us to fully understand the advantages and disadvantages of different algorithms.

We follow the standard terminology in the literature to call the nodes with known positions *anchors* and the node to be localized the *target node*. Bold upper case symbols denote matrices and bold lower case symbols denote vectors. The **0**_*m*×*n*_ is the *m*×*n* zero matrix and **I**_*m*_ is the *m*×*m* identity matrix. The transpose and 2-norm of a vector **x** are denoted by (·)^*T*^ and ∥**x**∥, respectively. For two symmetric matrices **A** and **B**, **A**≽**B** means that **A** **−** **B** is positive semidefinite.

## System model

An ATDOA localization system consists of a number of nodes. The anchor node that initiates the pulse transmission is called anchor Tx, and the one that receives the pulse is called anchor Rx. In an ATDOA system, there are multiple anchor Tx nodes and one anchor Rx node.

Let **x**=[*x*,*y*]^*T*^ and **x**_*i*_=[*x*_*i*_,*y*_*i*_]^*T*^,*i*=1,2,…,*M* be the coordinates of the target node and anchor nodes, respectively, where *M* is the number of anchor nodes with *M*≥3 for two-dimensional positioning. Without loss of generality, let anchor Rx be at **x**_1_, and anchor Tx be positioned at **x**_*i*_, *i*=2,3,…,*M*.

**Fig. 1:**
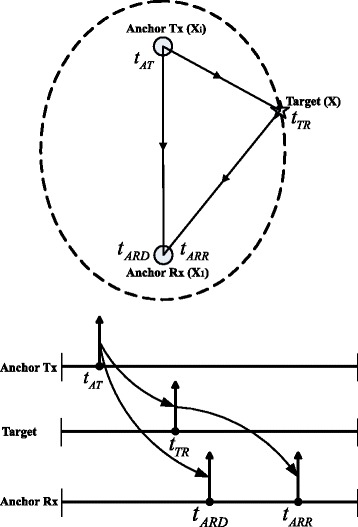
A-TDOA localization system signal flow and timing diagram

Figure 1 demonstrates the signal flow and the system timing. At time *t*_*AT*_, anchor Tx transmits a pulse that is received at the target node at time *t*_*TR*_ and at anchor Rx at time *t*_*ARD*_. As soon as the target node receives the pulse from anchor Tx, it re-transmits it immediately. The re-transmitted signal then reaches anchor Rx at time *t*_*ARR*_. Ultimately, anchor Rx will receive two pulses in a row: one is from anchor Tx, and the other is from the target node.

The time difference measured at anchor Rx can be written as

1 $$   \begin{aligned} \left(t_{ARR} - t_{ARD} \right) \cdot c &= \left\| \textbf{x} - \textbf{x}_{i} \right\| + \left\| {\textbf{x} - {\textbf{x}_{1}}} \right\| - \left\| {{\textbf{x}_{i}} - {\textbf{x}_{1}}} \right\| + {n_{i}},\\ i &= 2,3,\ldots,M, \end{aligned}  $$

where *n*_*i*_ is a zero mean measurement error. Equation (1) exhibits the beauty of the ATDOA system that the time difference (*t*_*ARR*_−*t*_*ARD*_) is measured at and only at anchor Rx. Therefore, no clock synchronization is required among anchor Rx, anchor Tx and the target node. The use of the backbone cables which are mandatory in conventional TDOA positioning systems can now be avoided.

**Fig. 2:**
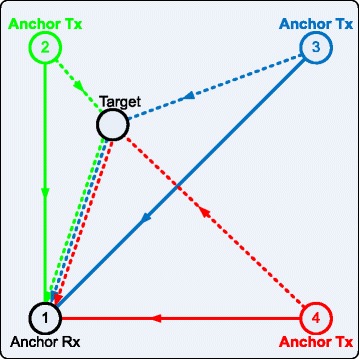
An example of an ATDOA system layout

An example system layout is shown in Fig. 2. Three anchor Tx nodes and one anchor Rx node constitute the infrastructure. The solid lines indicate the direct radio paths between the anchor Tx nodes and the anchor Rx node, and the dashed lines indicate the re-transmitted radio paths. The ATDOA localization system can be made self-contained when all anchor nodes are equipped with data communication capability. A communication protocol is also necessary to coordinate the localization measurements sequence. A complete location measurement cycle involves several TDOA ranging measurements, and each measurement takes place by having one anchor Tx emits a pulse, and the anchor Rx measures the TDOA. The anchor Rx coordinates the sequence by signaling each anchor Tx in order. Once a specific anchor Tx receives the signaling from the anchor Rx, it responses its anchor identification and location, followed by a ranging pulse. The data communication and protocol can be implemented in numerous ways, which are beyond the scope of this work. In general, with *M* anchor nodes, there are *M*(*M*−1)/2 distinct ATDOA measurements from all anchor node pairs. We shall call any set of (*M*−1) measurements a non-redundant set. In the noiseless case, a non-redundant set is sufficient to determine the exact target node position. Nevertheless, noise and estimation errors are unavoidable in real-world problems. As will be seen in Section 3, a proper selection of a non-redundant set can significantly improve the estimation accuracy in an ATDOA system by applying receiver re-selection technique.

## Cramer-Rao lower bound

Cramer-Rao lower bound is commonly used for providing a lower bound on an estimator’s mean square error (MSE). It establishes a fundamental limit on the achievable localization accuracy, and it serves as a benchmark for any unbiased location estimator. Previous works derive CRLB based on modeling range estimates as being corrupted by zero mean Gaussian noise [22, 23]. These works made an assumption that the variance of the range estimate is not dependent on the actual node pair distance. As a matter of fact, signal power decays as the propagation distance increases in practical situation. In an indoor environment, the path loss exponent can vary from 2 to 6 [24], and the signal power decays 20 to 60 dB as the propagation distance increases by a decade. This results in a significantly received signal power variation. Given a constant thermal noise level, received signal power variation results in a change in SNR, which in turn determines the achievable localization accuracy [23]. To reflect the SNR change, we follow a similar approached used in [25] to model noise variance as a distance dependent parameter. Such modeling is applied throughout this paper. Below, we derive a distance-dependent CRLB for the ATDOA system.

Firstly, we denote the measured distance difference between a direct path and a re-transmitted path as

2 $$  r_{i} = d_{i} + n_{i}, \:\: i = 2, 3, \ldots, M,  $$

where *d*_*i*_ is the true distance difference of arrival

3 $$  d_{i} = \left\| \textbf{x} - \textbf{x}_{i} \right\| + \left\| \textbf{x} - \textbf{x}_{1} \right\| - \left\| \textbf{x}_{i} - \textbf{x}_{1} \right\|  $$

and $n_{i} \sim \mathcal {N}(0,\sigma _{i}^{2})$ is a zero mean Gaussian error, whose variance is modeled as

4 $$  \sigma_{i}^{2} = K_{E}\left(\left\| \textbf{x} - \textbf{x}_{i} \right\| + \left\| \textbf{x} - \textbf{x}_{1} \right\| \right)^{\beta} + K_{E}\left(\left\| \textbf{x}_{i} - \textbf{x}_{1} \right\| \right)^{\beta}.  $$

In (4), *K*_*E*_ is a proportionality constant to capture the combined physical layer effect on the range estimate, and *β* is the path loss exponent. Compared to TOA noise variance, the ATDOA system’s noise variance is significantly higher, due to that the extra signal transmission scheme is involved. We start by writing the probability density function for *r*_*i*_ as

5 $$  f\left(r_{i}| d_{i}\right) = \frac{1}{\sqrt{2\pi \sigma_{i}^{2}}}\exp \left(- \frac{\left(r_{i} - d_{i} \right)^{2}}{2\sigma_{i}^{2}} \right).  $$

The log-likelihood function can then be expressed as

6 $$  {\begin{aligned} \ln f\left(r|\textbf{x} \right) &= - \frac{1}{2}\ln \left(2\pi {K_{E}} \right) \\ &\quad- \frac{1}{2}\ln \left[\left(\left\|\textbf{x} \,-\, \textbf{x}_{i} \right\| \,+\, \left\| \textbf{x} - \textbf{x}_{1} \right\| \right)^{\beta} + \left(\left\|\textbf{x}_{i}\! -\! \textbf{x}_{1} \right\| \right)^{\beta}\right] \\ &\quad- \frac{1}{2K_{E}}\frac{\left(\left\|\textbf{x} \,-\, \textbf{x}_{i} \right\| \,+\, \left\| \textbf{x} \,-\, \textbf{x}_{1} \right\| - \left\|\textbf{x}_{i} - \textbf{x}_{1} \right\| - r \right)^{2}}{\left(\left\| \textbf{x} - \textbf{x}_{i} \right\| + \left\| \textbf{x} - \textbf{x}_{1} \right\| \right)^{\beta} + \left(\left\|\textbf{x}_{i} \,-\, \textbf{x}_{1} \right\| \right)^{\beta}} \end{aligned}}  $$

For the sake of simpler expression, we denote

7 $$  A = - \frac{1}{2}\ln \left[ {{{\left({\left\| {\textbf{x} - {\textbf{x}_{{i}}}} \right\| + \left\| {\textbf{x} - {\textbf{x}_{{1}}}} \right\|} \right)}^{\beta}} + {{\left({\left\| {{\textbf{x}_{{i}}} - {\textbf{x}_{{1}}}} \right\|} \right)}^{\beta} }} \right],  $$

8 $$  B = - \frac{1}{{2{K_{E}}}}\frac{{{{\left({\left\| {\textbf{x} - {\textbf{x}_{{i}}}} \right\| + \left\| {\textbf{x} - {\textbf{x}_{{1}}}} \right\| - \left\| {{\textbf{x}_{{i}}} - {\textbf{x}_{{1}}}} \right\| - r} \right)}^{2}}}}{{{{\left({\left\| {\textbf{x} - {\textbf{x}_{{i}}}} \right\| + \left\| {\textbf{x} - {\textbf{x}_{{1}}}} \right\|} \right)}^{\beta}} + {{\left({\left\| {{\textbf{x}_{{i}}} - {\textbf{x}_{{1}}}} \right\|} \right)}^{\beta} }}}.  $$

According to [26], the CRLB is found as the [ i_*th*_,i_*th*_] element of the inverse of the Fisher information matrix (FIM). To derive the FIM, we calculate the second derivative of the likelihood function and then apply the expectation operation as:

9a $$\begin{array}{*{20}l} &\mathbb{E}\left[ {\frac{{{\partial^{2}}A}}{{\partial {{{x}}^{2}}}}} \right] + \mathbb{E}\left[ {\frac{{{\partial^{2}}B}}{{\partial {{{x}}^{2}}}}} \right]\\ &\quad= - \frac{{{\beta^{2}} \cdot {p_{i}}^{2\beta - 2} \cdot {f_{xxi}}}}{{2g_{i}^{2}}}- \frac{{\beta \cdot {p_{i}}^{\beta - 1} \cdot {s_{xxi}}}}{{2{g_{i}}}} - \frac{{{f_{xxi}}}}{{{K_{E}}{g_{i}}}} \end{array} $$

9b $$\begin{array}{*{20}l} & \mathbb{E}\left[ {\frac{{{\partial^{2}}A}}{{\partial {{{y}}^{2}}}}} \right] + \mathbb{E}\left[ {\frac{{{\partial^{2}}B}}{{\partial {{{y}}^{2}}}}} \right]\\ &\quad= - \frac{{{\beta^{2}} \cdot {p_{i}}^{2\beta - 2} \cdot {f_{yyi}}}}{{2g_{i}^{2}}} - \frac{{\beta \cdot {p_{i}}^{\beta - 1} \cdot {s_{yyi}}}}{{2{g_{i}}}} - \frac{{{f_{yyi}}}}{{{K_{E}}{g_{i}}}} \end{array} $$

9c $$\begin{array}{*{20}l} & \mathbb{E}\left[ {\frac{{{\partial^{2}}A}}{{\partial {{x}}\partial {{y}}}}} \right] + \mathbb{E}\left[ {\frac{{{\partial^{2}}B}}{{\partial {{x}}\partial {{y}}}}} \right]\\ &\quad= - \frac{{{\beta^{2}} \cdot {p_{i}}^{2\beta - 2} \cdot {f_{xyi}}}}{{2g_{i}^{2}}} - \frac{{\beta \cdot {p_{i}}^{\beta - 1} \cdot {s_{xyi}}}}{{2{g_{i}}}} - \frac{{{f_{xyi}}}}{{{K_{E}}{g_{i}}}} \end{array} $$

where

10a $$\begin{array}{*{20}l} &{g_{i}} = {\left({\left\| {\textbf{x} - {\textbf{x}_{{i}}}} \right\| + \left\| {\textbf{x} - {\textbf{x}_{{1}}}} \right\|} \right)^{\beta}} + {\left\| {{\textbf{x}_{{i}}} - {\textbf{x}_{{1}}}} \right\|^{\beta} } \end{array} $$

10b $$\begin{array}{*{20}l} & {f_{xxi}} = {\left({\frac{{x - {x_{1}}}}{{\left\| {\textbf{x} - {\textbf{x}_{{1}}}} \right\|}} + \frac{{x - {x_{i}}}}{{\left\| {\textbf{x} - {\textbf{x}_{{i}}}} \right\|}}} \right)^{2}} \end{array} $$

10c $$\begin{array}{*{20}l} & {f_{yyi}} = {\left({\frac{{y - {y_{1}}}}{{\left\| {\textbf{x} - {\textbf{x}_{{1}}}} \right\|}} + \frac{{y - {y_{i}}}}{{\left\| {\textbf{x} - {\textbf{x}_{{i}}}} \right\|}}} \right)^{2}} \end{array} $$

10d $$\begin{array}{*{20}l} & {f_{xyi}} = \left({\frac{{x - {x_{1}}}}{{\left\| {\textbf{x} - {\textbf{x}_{{1}}}} \right\|}} + \frac{{x - {x_{i}}}}{{\left\| {\textbf{x} - {\textbf{x}_{{i}}}} \right\|}}} \right)\\ &\qquad\times\left({\frac{{y - {y_{1}}}}{{\left\| {\textbf{x} - {\textbf{x}_{{1}}}} \right\|}} + \frac{{y - {y_{i}}}}{{\left\| {\textbf{x} - {\textbf{x}_{{i}}}} \right\|}}} \right) \end{array} $$

10e $$\begin{array}{*{20}l} & {p_{i}} = \left\| {\textbf{x} - {\textbf{x}_{{i}}}} \right\| + \left\| {\textbf{x} - {\textbf{x}_{{1}}}} \right\| \end{array} $$

10f $$\begin{array}{*{20}l} & {s_{xxi}} = \frac{{{{\left({x - {x_{1}}} \right)}^{2}}}}{{{{\left\| {\textbf{x} - {\textbf{x}_{{1}}}} \right\|}^{3}}}} + \frac{{{{\left({x - {x_{i}}} \right)}^{2}}}}{{{{\left\| {\textbf{x} - {\textbf{x}_{{i}}}} \right\|}^{3}}}} - \frac{1}{{\left\| {\textbf{x} - {\textbf{x}_{{1}}}} \right\|}} - \frac{1}{{\left\| {\textbf{x} - {\textbf{x}_{{i}}}} \right\|}} \end{array} $$

10g $$\begin{array}{*{20}l} & {s_{yyi}} = \frac{{{{\left({y - {y_{1}}} \right)}^{2}}}}{{{{\left\| {\textbf{x} - {\textbf{x}_{{1}}}} \right\|}^{3}}}} + \frac{{{{\left({y - {y_{i}}} \right)}^{2}}}}{{{{\left\| {\textbf{x} - {\textbf{x}_{{i}}}} \right\|}^{3}}}}- \frac{1}{{\left\| {\textbf{x} - {\textbf{x}_{{1}}}} \right\|}} - \frac{1}{{\left\| {\textbf{x} - {\textbf{x}_{{i}}}} \right\|}} \end{array} $$

10h $$\begin{array}{*{20}l} & {s_{xyi}} = \frac{{\left({x - {x_{1}}} \right)\left({y - {y_{1}}} \right)}}{{{{\left\| {\textbf{x} - {\textbf{x}_{{1}}}} \right\|}^{3}}}} + \frac{{\left({x - {x_{i}}} \right)\left({y - {y_{i}}} \right)}}{{{{\left\| {\textbf{x} - {\textbf{x}_{{i}}}} \right\|}^{3}}}}\\ &\qquad - \frac{1}{{\left\| {\textbf{x} - {\textbf{x}_{{1}}}} \right\|}} - \frac{1}{{\left\| {\textbf{x} - {\textbf{x}_{{i}}}} \right\|}}. \end{array} $$

Ultimately, the elements in the FIM can be written as:

11a $$\begin{array}{*{20}l} &{\left[ {F\left(\textbf{x} \right)} \right]_{11}} = \sum\limits_{i = 1}^{N} \left[ \frac{{{\beta^{2}} \cdot {p_{i}}^{2\beta - 2} \cdot {f_{xxi}}}}{{2g_{i}^{2}}} + \frac{{\beta \cdot {p_{i}}^{\beta - 1} \cdot {s_{xxi}}}}{{2{g_{i}}}}\right.\\ &\qquad\quad\qquad\left.+ \frac{{{f_{xxi}}}}{{{K_{E}}{g_{i}}}} {\vphantom{\frac{{{\beta^{2}} \cdot {p_{i}}^{2\beta - 2} \cdot {f_{yyi}}}}{{2g_{i}^{2}}}}}\right] \end{array} $$

11b $$\begin{array}{*{20}l} &{\left[ {F\left(\textbf{x} \right)} \right]_{22}} = \sum\limits_{i = 1}^{N} \left[ \frac{{{\beta^{2}} \cdot {p_{i}}^{2\beta - 2} \cdot {f_{yyi}}}}{{2g_{i}^{2}}} + \frac{{\beta \cdot {p_{i}}^{\beta - 1} \cdot {s_{yyi}}}}{{2{g_{i}}}}\right.\\ &\left.\qquad\quad\qquad+ \frac{{{f_{yyi}}}}{{{K_{E}}{g_{i}}}}{\vphantom{\frac{{{\beta^{2}} \cdot {p_{i}}^{2\beta - 2} \cdot {f_{yyi}}}}{{2g_{i}^{2}}}}} \right] \end{array} $$

11c $$\begin{array}{*{20}l} &{\left[ {F\left(\textbf{x} \right)} \right]_{12}} = {\left[ {F\left(\textbf{x} \right)} \right]_{21}} = \sum\limits_{i = 1}^{N} \left[ \frac{{{\beta^{2}} \cdot {p_{i}}^{2\beta - 2} \cdot {f_{xyi}}}}{{2g_{i}^{2}}}\right.\\ &\qquad\quad\qquad\qquad\qquad\quad\left.+ \frac{{\beta \cdot {p_{i}}^{\beta - 1} \cdot {s_{xyi}}}}{{2{g_{i}}}} + \frac{{{f_{xyi}}}}{{{K_{E}}{g_{i}}}} \right]. \end{array} $$

Typically, the constant *K*_*E*_ is extremely small and therefore the third terms in (11), i.e., $\frac {{{f_{xxi}}}}{{{K_{E}}{g_{i}}}}$, $\frac {{{f_{yyi}}}}{{{K_{E}}{g_{i}}}}$ and $\frac {{{f_{xyi}}}}{{{K_{E}}{g_{i}}}}$ dominates.

**Fig. 3:**
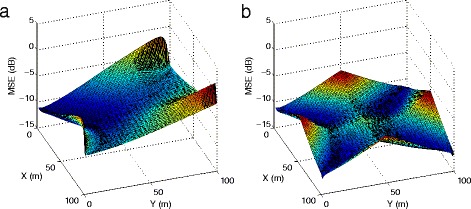
**a** The CRLB of an ATDOA localization system without receiver re-selection. **b** The CRLB of an ATDOA localization system with receiver re-selection

The CRLB of an ATDOA system is shown in Fig. 3 a. The X- and Y- axis indicate the target node’s coordinate, and the Z- axis is the mean square position error expressed in dB (for instance, -20 dB corresponds to $\sqrt {10^{-20/10}} = 0.1 \text {~m}$). The target node’s estimation error is evaluated at each coordinate in a 100×100 m area. The anchor Rx is placed at (0, 0), and three anchor Tx nodes are located at (0, 100), (100, 0), and (100, 100). The path loss exponent *β* is set to 4 to capture a realistic radio propagation channel. The constant *K*_*E*_ is set to $\frac {\sigma _{0}^{2}}{(50\sqrt {2})^{\beta }}$, so that when the target node is at the center, i.e., coordinate (50, 50), the noise variance from the target node to any anchor node is $K_{E} \cdot \left \| {\textbf {x} - {\textbf {x}_{{i}}}} \right \|^{\beta } = \frac {\sigma _{0}^{2}}{(50\sqrt {2})^{\beta }} \cdot (50\sqrt {2})^{\beta } = \sigma _{0}^{2}$. We used *σ*_0_=0.1 m in the simulation. It is obvious in Fig. 3 a that the position estimation error close to the anchor Rx is much smaller than other positions, largely due to that the noise variance is smaller when the target node is close to the anchor Rx. Therefore, when multiple transceiver anchor nodes are available in the system, the anchor Rx node can be chosen as the one closest to the target node to minimize the estimation error. We refer to this method as “receiver re-selection”. This method requires the system to have a priori knowledge of an approximate target node position. This a priori knowledge can easily be obtained by using a localization algorithm that achieves global convergence to estimate approximate coordinates of the target node. This position estimate can then be used to re-select the receiver node. Given the updated receiver node and the approximate target node coordinates as an initial guess, a high accuracy algorithm can then be applied to give superb performance. Figure 3 b demonstrates the improved CRLB by selecting a proper anchor Rx such that the CRLB becomes minimal.

**Fig. 4:**
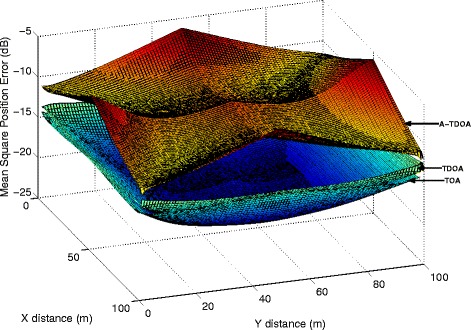
Comparison of the TOA, TDOA, and ATDOA system CRLB

We also derive the distance-dependent CRLB for TOA and TDOA systems, and a comparison of them is shown in Fig. 4. It is observed that the TOA system achieves the lowest MSE, which is less than –14 dB (approximately 0.2 m). The TDOA system’s MSE is about 1 dB higher than TOA system. It is obvious that the ATDOA CRLB is about 8 dB higher than the TOA CRLB. This is largely due to the extra signal transmission scheme involved in the localization process, that is, it requires both direct path and re-transmitted path signal for localization, and the compound noise power is significantly higher than TOA and TDOA systems. Nevertheless, although the ATDOA system signaling is slightly complicated and the performance is poorer, it worths the effort to relax the more difficult clock synchronization requirement and therefore provides great potential for practical use.

## A high accuracy two-step localization algorithm

In this section, we propose a two-step localization algorithm that combines a SDP technique and a Taylor series method to achieve high estimation accuracy. Typically, SDP is used to relax the non-convex problem to a convex problem so as to obtain a global estimation of the true position regardless of the initial point used [27]. The solution is then used as an initial guess for the Taylor series method to achieve superior performance. In addition, as the SDP method can achieve global minimal, if necessary, the estimated target position can be used to re-select the anchor Rx node to minimize the estimation error. Below, we derive the two-step localization algorithm.

The ML estimator for the A-TDOA system can be obtained as

12 $$  \arg \mathop {\min }\limits_{\textbf{x}} \sum\limits_{i = 2}^{M} {\frac{{{{\left({{r_{i}} - \left\| {\textbf{x} - {\textbf{x}_{i}}} \right\| - \left\| {\textbf{x} - {\textbf{x}_{1}}} \right\| + \left\| {{\textbf{x}_{i}} - {\textbf{x}_{1}}} \right\|} \right)}^{2}}}}{{\sigma_{i}^{2}}}}.  $$

Equation (12) is highly non-linear and non-convex, hence, an improper selection of the initial guess may lead to a local convergence which is deviated from a global minimal. Next, we will be using SDP to relax the non-convex optimization problem to a convex optimization problem to provide approximate position estimation in a globally optimum fashion [27]. We start by expanding (12) as

13 $$  {\begin{aligned} \arg \mathop {\min }\limits_{\textbf{x}} \sum\limits_{i = 2}^{M} & {\frac{{{{\left\| {\textbf{x} - {\textbf{x}_{i}}} \right\|}^{2}} + 2 \cdot \left\| {\textbf{x} - {\textbf{x}_{i}}} \right\| \cdot \left\| {\textbf{x} - {\textbf{x}_{1}}} \right\| + {{\left\| {\textbf{x} - {\textbf{x}_{1}}} \right\|}^{2}}}}{{\sigma_{i}^{2}}}} \\ & - \frac{{2 \cdot \left\| {\textbf{x} - {\textbf{x}_{i}}} \right\|\left({\left\| {{\textbf{x}_{i}} - {\textbf{x}_{1}}} \right\| + {r_{i}}} \right) + 2 \cdot \left\| {\textbf{x} - {\textbf{x}_{1}}} \right\|\left({\left\| {{\textbf{x}_{i}} - {\textbf{x}_{1}}} \right\| + {r_{i}}} \right)}}{{\sigma_{i}^{2}}} \\ & + \frac{{r_{i}^{2} + {{\left\| {{\textbf{x}_{i}} - {\textbf{x}_{1}}} \right\|}^{2}} + 2{r_{i}}\left\| {{\textbf{x}_{i}} - {\textbf{x}_{1}}} \right\|}}{{\sigma_{i}^{2}}}. \end{aligned}}  $$

To convert the non-convex quadratic distance constraints into convex constraints, we introduce a relaxation to remove the quadratic terms in the formulation to convert problem (13) into a standard SDP problem as Eq. (14) (see Appendix).

14a $$\begin{array}{*{20}l} &\arg \mathop {\min }\limits_{\textbf{x},\textbf{h},\textbf{R},{{z}}} \sum\limits_{i = 2}^{M} {\frac{{{h_{ii}} + 2 \cdot {h_{i1}} + {h_{11}}}}{{\sigma_{i}^{2}}}}  \\ & - \frac{{2 \cdot {h_{i}}\left({\left\| {{\textbf{x}_{i}} - {\textbf{x}_{1}}} \right\| + {r_{i}}} \right) + 2 \cdot {h_{1}}\left({\left\| {{\textbf{x}_{i}} - {\textbf{x}_{1}}} \right\| + {r_{i}}} \right)}}{{\sigma_{i}^{2}}}  \end{array} $$

14b $$\begin{array}{*{20}l} & \mbox {subject to:}  \\ & \left[\begin{array}{ll} \mathbf{H}&\mathbf{h}\\ \mathbf{h}^{T}&1 \end{array}\right] \succeq \mathbf{0}_{(m+1)\times(m+1)}  \end{array} $$

14c $$\begin{array}{*{20}l} & \left[\begin{array}{ll} z&\mathbf{x}^{T} \\ \mathbf{x}&\mathbf{I}_{2} \end{array}\right] \succeq \mathbf{0}_{3\times 3}  \end{array} $$

14d $$\begin{array}{*{20}l} & h_{ii} = z + \mathbf{x}_{i}^{T}\mathbf{x}_{i} - 2\mathbf{x}_{i}^{T}\mathbf{x}, i = 1, 2, \dots, M,  \end{array} $$

In minimizing the objective function in (14a), *h*_*i*1_ tends to decrease while *h*_*i*_ and *h*_1_ tend to increase, hence, the relaxation made above is not tight. Nevertheless, (14) is a convex problem whose global solution can readily be computed. In addition, simulation studies have indicated that the approximate solution to problem (14) is typically close to the true location. Based on these, we propose a two-step algorithm in that the SDP solution serves as an initial estimation to allow a Taylor-series-based method step a quick convergence to an accurate location estimation.

To describe the Taylor-series-based approach, we denote *f*_*i*_(**x**)=∥**x**−**x**_*i*_∥+∥**x**−**x**_1_∥ and express the error function as

15 $$  e_{i}(\mathbf{x}) = r_{i} + \Arrowvert \mathbf{x}_{i} - \mathbf{x}_{1} \Arrowvert - f_{i}(\mathbf{x}), i = 2, 3, \dots, M.  $$

Let **x**_0_= [ *x*_0_,*y*_0_]^*T*^ be the initial guess of the target location and *Δ* **x**= [ *δ* *x*,*δ* *y*]^*T*^ be the small increment on **x**.

By applying Taylor expansion to the equations in (15), it can be linearized to

16 $$   e_{i}(\mathbf{x}) \approx r_{i} + \Arrowvert \mathbf{x}_{i} - \mathbf{x}_{1} \Arrowvert - f_{i}(\mathbf{x}_{0}) - \frac{\partial f_{i}(\mathbf{x})}{\partial x} \bigg|_{\mathbf{x}_{0}} \cdot \delta x - \frac{\partial f_{i}(\mathbf{x})}{\partial y} \bigg|_{\mathbf{x}_{0}} \cdot \delta y,  $$

which can be expressed in vector form as

17 $$  \mathbf{e} = \mathbf{b - A \cdot \Delta \mathbf{x}},  $$

where

18 $$  \mathbf{A} \triangleq \left[ \begin{array}{cc} \frac{\partial f_{2}(\mathbf{x})}{\partial x} & \frac{\partial f_{2}(\mathbf{x})}{\partial y} \\ \frac{\partial f_{3}(\mathbf{x})}{\partial x} & \frac{\partial f_{3}(\mathbf{x})}{\partial y} \\ \vdots & \vdots \\ \frac{\partial f_{M}(\mathbf{x})}{\partial x} & \frac{\partial f_{M}(\mathbf{x})}{\partial y} \end{array} \right],  $$

19 $$  \mathbf{b} \triangleq \left[ \begin{array}{c} r_{2} + \Arrowvert \mathbf{x}_{2} - \mathbf{x}_{1} \Arrowvert - f_{2}(\mathbf{x}_{0}) \\ r_{3} + \Arrowvert \mathbf{x}_{3} - \mathbf{x}_{1} \Arrowvert - f_{3}(\mathbf{x}_{0}) \\ \vdots \\ r_{M} + \Arrowvert \mathbf{x}_{M} - \mathbf{x}_{1} \Arrowvert - f_{M}(\mathbf{x}_{0}) \\ \end{array} \right].  $$

The least-squares estimate for (17) is given by

20 $$  {\Delta \mathbf{x} = \mathbf{(A^{T}W^{-1}A)^{-1}A^{T}Wb},}  $$

where **W** is the weight matrix $ \mathbf {W} = \text {diag} \left \{ \sigma _{2}^{2}, \sigma _{3}^{2}, \cdots, \sigma _{M}^{2} \right \}$. The target location is then updated to

21 $$  \mathbf{x} = \mathbf{x}_{0} + \Delta \mathbf{x}.  $$

The updated target location is utilized in the next iteration until the magnitude of *Δ* **x** becomes less than a prescribed tolerance. It is reasonable to treat the measurement error variance $\sigma _{i}^{2}$ as a known value in both SDP and Taylor steps, because modern receiver is capable of measuring signal-to-noise ratio which is inversely related to the $\sigma _{i}^{2}$. Simulation results and analysis of the two-step estimator are provided in Section 6.

## A low complexity constrained least-squares localization algorithm

The two-step algorithm presented in Section 4 provides an accurate solution at the cost of a relatively high computational complexity. As an alternative solution, in this section, we present a constrained least-squares estimator providing good accuracy with reduced complexity. We start by rewriting the error functions in (15) as

22a $$ \hat{\mathbf{e}}(\mathbf{x}) = \mathbf{Bt - q},   $$

where

22b $$  \mathbf{B } = \left[ \begin{array}{ccccc} 1 & 1 & 0 & \ldots & 0 \\ 1 & 0 & 1 & \ldots & 0 \\ \vdots & \vdots & \vdots & \ddots & \vdots \\ 1 & 0 & 0 & \ldots & 1 \end{array} \right]_{(M-1)\times M}, \mathbf{t} = \left[ \begin{array}{c} t_{1}\\ t_{2}\\ \vdots \\ t_{M} \end{array} \right], \mathbf{q} = \left[ \begin{array}{c} q_{2}\\ q_{3}\\ \vdots \\ q_{M} \end{array} \right],   $$

with *t*_*i*_=∥**x**−**x**_*i*_∥, *q*_*i*_=*r*_*i*_+∥**x**_*i*_−**x**_1_∥ and *r*_*i*_ representing the measured range differences.

The localization problem at hand can be formulated as a constrained least-squares problem

23a $$\begin{array}{*{20}l} &\min_{\mathbf{x,r}} \Arrowvert \mathbf{Bt - q} \Arrowvert^{2}  \end{array} $$

23b $$\begin{array}{*{20}l} & \mbox {subject to:}  \\ & t_{i} = \Arrowvert \mathbf{x} - \mathbf{x}_{i} \Arrowvert, i= 1, 2, \cdots, M.  \end{array} $$

Let the singular value decomposition [28] of matrix **B** be given by

24 $$  \mathbf{B} = \mathbf{U}\mathbf{\Sigma}\mathbf{V}^{T},  $$

where $\mathbf {U} \in \ \mathbf {\mathcal {R}}^{(M-1)\times (M-1)}$ and $\mathbf {V} \in \mathbf {\mathcal {R}}^{M \times M}$ are orthogonal and **Σ**= [ **S** **0**] with **S**=diag{*λ*_1_,*λ*_2_,⋯,*λ*_*M*−1_}>**0**. Using (24), we can write

25 $$  \Arrowvert \mathbf{Bt - q} \Arrowvert = \Arrowvert \mathbf{\Sigma}\mathbf{z} - \tilde{\mathbf{q}} \Arrowvert  $$

where **z**=**V** ^*T*^ **t** and $\tilde {\mathbf {q}} = \mathbf {U}^{T}\mathbf {q}$. If we denote

26 $$  \mathbf{z} = \left(\begin{array}{c} \mathbf{\hat{z}}\\ \phi \end{array} \right),  $$

where *ϕ* is a free-scaler parameter, then (25) becomes $\Arrowvert \mathbf {Bt - q} \Arrowvert = \Arrowvert \mathbf {S} \hat {\mathbf {z}}-\tilde {\mathbf {q}} \Arrowvert $, hence ∥**B** **t** **−** **q**∥ reaches its minimum if $\hat {\mathbf {z}} = \mathbf {S^{-1}}\tilde {\mathbf {q}}$, and the optimal **z** is given by

27 $$  \mathbf{z^{\ast}} = \left(\begin{array}{c} \mathbf{S^{-1}}\tilde{\mathbf{q}}\\ \phi \end{array} \right),  $$

Therefore, the optimal **t** for (23a) is given by

28 $$  \begin{aligned} \mathbf{t^{\ast}} &= \mathbf{Vz^{\ast}} = \left[\underbrace{\mathbf{V_{1}}}_{M-1} \; \underbrace{v_{M}}_{1}\right] \left[ \begin{array}{c} \mathbf{S^{-1}}\tilde{\mathbf{q}} \\ \phi \end{array} \right] = \mathbf{V_{1}S^{-1}}\mathbf{U}^{T}\mathbf{q}\\ &\quad+ v_{M}\phi \triangleq \mathbf{t_{s}} + v_{M}\phi, \end{aligned}  $$

where parameter *ϕ* will be optimally tuned in the next step in dealing with (23b). With the optimal **t** determined in (28), the constraints in (23b) become as

29 $$  \mathbf{h(x)} - v_{M}\phi - \mathbf{t_{s}} = 0,  $$

where

30 $$  \mathbf{h(x)} = \left[ \begin{array}{c} \Arrowvert \mathbf{x} - \mathbf{x}_{1} \Arrowvert \\ \Arrowvert \mathbf{x} - \mathbf{x}_{2} \Arrowvert \\ \vdots \\ \Arrowvert \mathbf{x} - \mathbf{x}_{M} \Arrowvert \end{array} \right].  $$

Hence, an *L*_2_-optimal approximate solution of (29) can be obtained by solving

31 $$  \min_{\mathbf{x},\phi} b(\mathbf{x},\phi) = \frac{1}{2} \Arrowvert \mathbf{h}(\mathbf{x}) - v_{M}\phi - \mathbf{t_{s}} \Arrowvert^{2}.  $$

The Gauss-Newton iteration [29] for minimizing *b*(**x**,*ϕ*) is given by

32 $$  \left[ \begin{array}{c} \mathbf{x}^{k+1}\\ \phi^{k+1} \end{array} \right] = \left[ \begin{array}{c} \mathbf{x}^{k}\\ \phi^{k} \end{array} \right] - \alpha_{k} \cdot \mathbf{H}^{-1}(\mathbf{x}_{i}) \cdot \nabla b(\mathbf{x}^{k},\phi^{k})  $$

where *α*_*k*_ is determined by an inexact line search, and

33 $$  \nabla b(\mathbf{x}^{k},\phi^{k}) = \mathbf{J}^{T}(\mathbf{x}^{k}) \cdot (h(\mathbf{x}^{k}) - v_{M}\phi^{k} - \mathbf{t}_{s})  $$

34 $$  \begin{aligned} \mathbf{H}(\mathbf{x}^{k}) &= \mathbf{J}^{T}(\mathbf{x}^{k}) \mathbf{J}(\mathbf{x}^{k}) + \epsilon \cdot \mathbf{I} \textrm{ with } \epsilon\\ &\quad\textrm{ a small positive constant, and} \end{aligned}  $$

35 $$  \mathbf{J}(\mathbf{x}) = \left[ \begin{array}{c|c} \frac{\mathbf{x}-\mathbf{x}_{1}}{\Arrowvert \mathbf{x} - \mathbf{x}_{1} \Arrowvert} & \\ \vdots & -v_{M} \\ \frac{\mathbf{x}-\mathbf{x}_{M}}{\Arrowvert \mathbf{x} - \mathbf{x}_{M} \Arrowvert} &\end{array} \right].  $$

We remark that matrix **B** in (22b) is independent of measurements, hence, *V*_1_ **,** **S** **,** **U** and *v*_*M*_ can be pre-calculated; and for two-dimensional location problems **H**(**x** ^*k*^) is of size 3×3, hence, the complexity of computing **H** ^−1^(**x** ^*k*^) as required in (32) is insignificant. The algorithm is found insensitive to its initial point $[\mathbf {x}_{0}^{T} \; \phi _{0}]^{T}$ as long as it is a reasonable one, e.g., $\mathbf {x}_{0} = \frac {1}{M}\sum _{i=1}^{M} \mathbf {x}_{i}$ and *ϕ*_0_=0. Typically, the algorithm converges in less than five iterations. Simulation results of the CLS algorithm and a detailed comparison with other estimators are presented in Section 6.

## Simulation results

Computer simulations have been conducted to corroborate the theoretical development and to evaluate the performance of the two-step and the CLS estimators. Four algorithms, namely, the two-step algorithm, the CLS algorithm, the linear least squares algorithm [11], as well as the SDP algorithm are compared. In addition, a comparison to the CRLB is provided to showcase the great accuracy achieved.

We adopted a consistent system geometry as shown in Section 2, with four anchor nodes placed at the vertex of a square, i.e., at (0, 0) m, (0, 100) m, (100, 100) m, and (100, 0) m. To fully evaluate the performance of the estimators, the target node is set to sweep a 100×100 m grid with a step size of 1 m moving towards either *X* or *Y* direction. The starting location is (0, 0) m, and the stopping location is (100, 100) m. To solve the SDP problem involved, the convex solver CVX [30] is applied. The initial guess point of the CLS algorithm is set to the mean value of the anchor nodes coordinate $\frac {1}{4} \sum _{k=1}^{4} \mathbf {x}_{k}$. Receiver re-selection technique is applied in all simulations to achieve the best possible performance. MSE is employed as the performance measure.

Measurement error was assumed to be Gaussian distributed with zero mean. By conducting extensive simulation, we observed that the performance of each estimator varies significantly depending on the measurement error variance. Therefore, we selected three typical error standard deviations, i.e., *σ*_0_=0.1 m, *σ*_0_=1 m and *σ*_0_=10 m to study each estimator’s performance under different conditions, where *σ*_0_ is the error standard deviation when the target node is at the center of the square, i.e., at (50, 50) m. Note that the measurement error model is still distance-dependent based on (4), and is treated as a known value. In real applications, a relative ranging error is usually more significant than an absolute error. For instance, 0.1 m ranging error in a 1 m distance measurement is considered inaccurate, while the same ranging error in a 100 m distance measurement is considered highly accurate. Therefore, we define a relative error percentage $P_{e} = \frac {\sigma }{d_{0}} \times 100\%$, where *d*_0_ is the distance between an anchor to the center of the measurement area, which in our layout is $d_{0} = 50 \sqrt {2}$. These three error magnitudes represent three typical real-life scenarios:

- Low ranging error (*σ*_0_≤0.1m): the ranging error is within ±0.2 m in 95% of the time. The relative error percentage is $P_{e} = \frac {0.1}{50\sqrt {2}} \cdot 100\%= 0.14\% $. Such high ranging accuracy is rarely reported in literature. It was only achieved in very carefully controlled experiment environments where high cost and high precision lab instruments were employed [31–33].
- Medium ranging error (0.1 m<*σ*_0_<10 m): the relative error percentage is within 0.14 to 14%. Most published works using TOA, TDOA, and two-way ranging techniques fall within this range [34–39]. The ATDOA system belongs to this category as well.
- High ranging error (*σ*_0_≥10 m): the relative error percentage is greater than 14%. Many system employing RSS ranging method fits in this category [40–42].

To the best of the author’s knowledge, there were no other works that thoroughly study the localization algorithm performance according to practical achievable ranging accuracy. This analysis method allows us to fully understand the advantages and disadvantages of each estimators, and hence, is of great importance to guide the selection of the algorithms in a reallife system. From Section 6.1 to Section 6.3, simulated performance of each algorithms under the aforementioned three scenarios are presented. Section 6.4 provides a comparison of algorithms with varying error magnitudes.

### Low ranging error simulation results

This section presents simulation results with an error standard deviation of 0.1 m in a 100×100 m area. The relative error percentage is extremely low, and such scenario is not very common in practical systems. Nevertheless, it well represents a system with a very high signal to noise ratio.

**Fig. 5:**
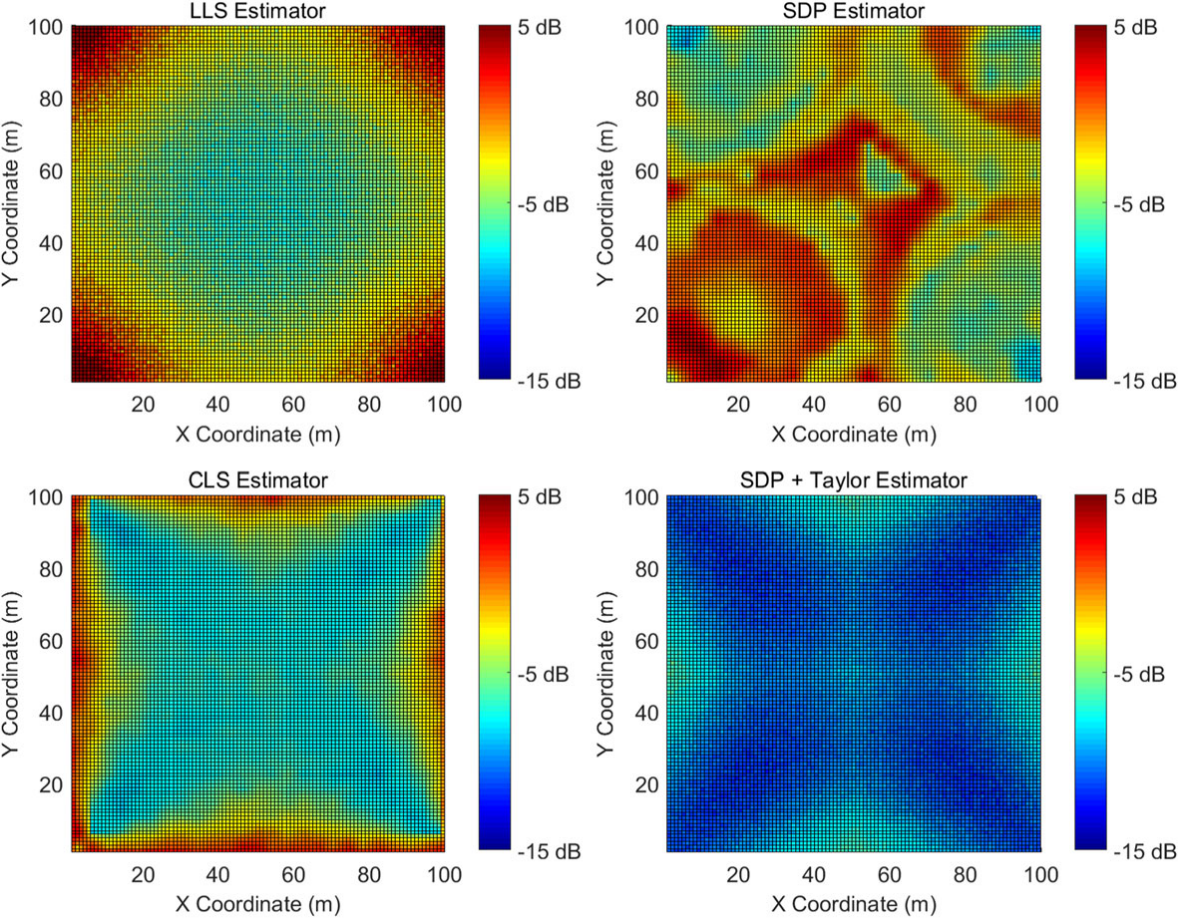
Simulated MSE with a ranging error of 0.1 m

Figure 5 demonstrates the simulated MSE for the LLS, SDP, CLS, and the two-step estimators. The X- and Y- axis define the position of the target node. Color-coded squares indicate the MSE of the estimators, and the cooler the color, the better the accuracy. All four sub-figures use the same color scale for easy comparison. In addition, Table 1 provides a summary of the MSE statistics for each estimators.

**Table 1:** Summary of the simulated MSE with a ranging error of 0.1 m

	Low ranging error simulation results summary			
Estimators	Mean (dB)	STD (dB)	Maximum (dB)	Minimum (dB)
LLS	−3.1	0.6	7.6	−8.1
SDP	−2.2	0.5	4.5	−9.9
CLS	−5.1	0.5	3.7	−9.1
Two-step	−10.1	0.3	−5.6	−12.3

It is very obvious in Fig. 5 that the two-step (SDP + Taylor) estimator outperforms all the other estimators. The average MSE over the entire 100×100m grid is several dB lower. In addition, the two-step estimator’s performance is rather consistent across the entire area. The CLS estimator has the second lowest MSE in all four estimators. Its average MSE is 5 dB higher than the two-step estimator, but 2 dB lower than the LLS estimator. Hence, the CLS estimator is a good compromise between the need for high accuracy and the demands of low complexity. The LLS estimator’s performance is reasonably satisfactory given it has the lowest complexity and an analytical solution. The SDP estimator performs the worst when the ranging error is low, yet we will find in Section 6.3 that it outperforms all the other algorithms when the ranging error is high. Another observation is that the MSE on the square edge is significantly higher than other positions for LLS, CLS, and the two-step estimators, and this is consistent with the CRLB shown in Section 3.

### Medium ranging error simulation results

This section presents simulation results with an error standard deviation of 1 m in a 100×100 m area. It well represents a practical system using time based localization techniques such as TOA, TDOA, and ATDOA.

**Fig. 6:**
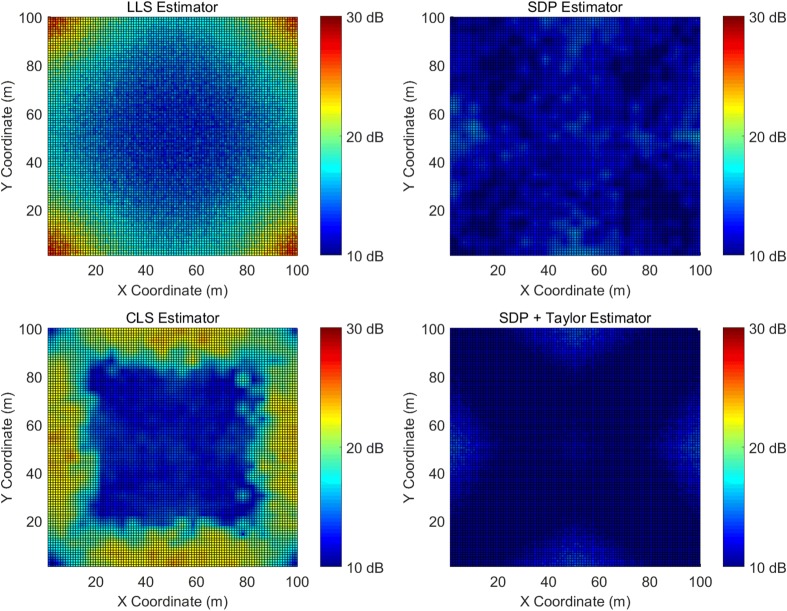
Simulated MSE with a ranging error of 1 m

Figure 6 demonstrates the simulated MSE for the LLS, SDP, CLS, and the two-step estimators and Table 2 provides a summary.

**Table 2:** Summary of the simulated MSE with a ranging error of 1 m

	Medium ranging error simulation results summary			
Estimators	Mean (dB)	STD (dB)	Maximum (dB)	Minimum (dB)
LLS	16.8	0.6	28.3	12.0
SDP	11.8	0.2	15.9	7.4
CLS	17.2	1.0	24.3	10.2
Two-step	10.0	0.2	14.3	6.9

Evidently, the two-step estimator still outperforms all the others and is still robust regardless of the target location. The SDP estimator performs the worst in low ranging error condition, however, its superiority is convincingly demonstrated as the error standard deviation increases to 1 m. There is only less than 2 dB difference between the SDP and the two-step estimator. The average MSE of the LLS, and the CLS estimators are comparable. The CLS provides a more accurate estimation in the center, while the LLS is generally better on the edge.

### High ranging error simulation results

This section presents simulation results with an error standard deviation of 10 m in a 100×100 m area, to study each estimators’ performance in a high relative error percentage condition, i.e., *P*_*e*_>14*%*. Although the ATDOA system generally has less than 14% relative percentage error, it is still worthwhile to study its performance under high ranging error condition. That is because the wireless channel varies with high dynamic range by shadowing and fading effects, which can cause the ranging accuracy to change significantly.

**Fig. 7:**
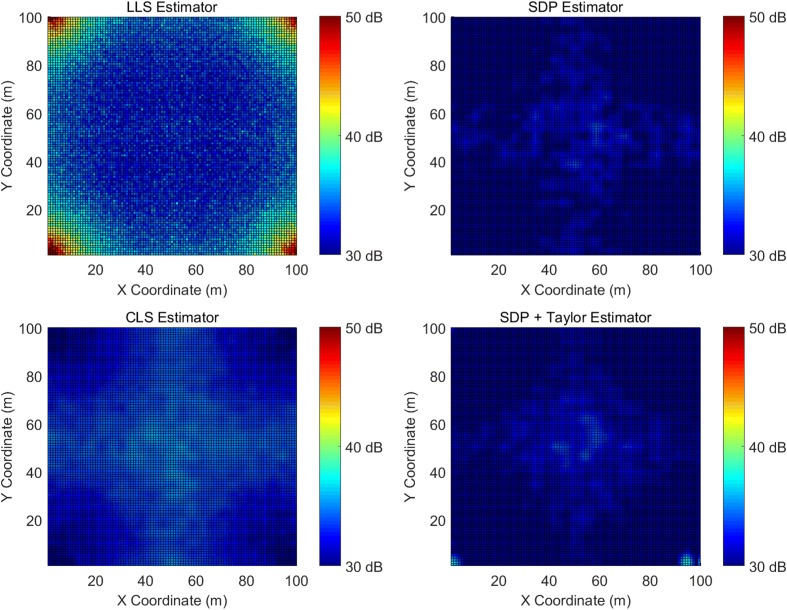
Simulated MSE with a ranging error of 10 m

Figure 7 demonstrates the simulated MSE for the LLS, SDP, CLS, and the two-step estimators and Table 3 provides a summary.

**Table 3:** Summary of the simulated MSE with a ranging error of 10 m

	Medium ranging error simulation results summary			
Estimator	Mean (dB)	STD (dB)	Maximum (dB)	Minimum (dB)
LLS	34.8	1.2	65.2	30.4
SDP	29.4	0.7	34.0	21.5
CLS	33.1	0.4	35.9	28.7
Two-step	30.0	0.3	38.6	26.9

As ranging error increases to 10 m, the SDP estimator’s average error is 0.6 dB less than the two-step estimator, and becomes the most accurate among all estimators. Although the two-step estimator is not the best performed under high ranging error condition, it still performs satisfactorily well. The CLS estimator performs consistently well regardless of the ranging error level. It has a low variation across the 100×100 m area, showcasing its strong robustness. The LLS estimator does not work well in high error condition. Its lowest estimation error is comparable to the maximum estimation error of the other estimators. Besides, its estimation error is particularly dependent on the target location.

### Estimation accuracy versus ranging error

Extensive simulations have been conducted to evaluate the performance of the two-step and the CLS algorithm under varying ranging errors and to compare their performance against the LLS, SDP, and the CRLB. Unlike Section 6.1 to Section 6.3, we fix the target node location and vary the ranging error magnitude, so we can compare their performance from a different angle.

**Fig. 8:**
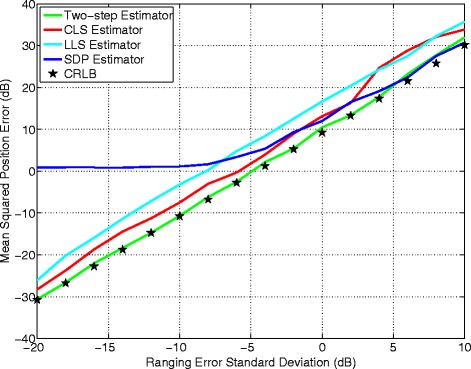
Algorithms comparison measured at (30 and 40 m)

**Fig. 9:**
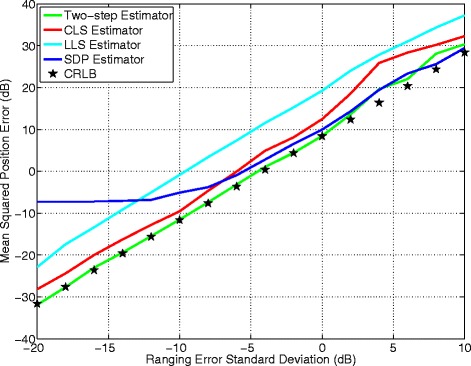
Algorithms comparison measured at (80 and 20 m)

Figures 8 and 9 depict the MSE versus ranging error with the target node located at (30, 40) m and (80, 20) m, respectively. It is observed that when the ranging error is relatively small, the two-step estimator closely follows the CRLB and outperforms the other estimators. When the ranging error becomes large, the two-step estimator’s performance is still highly satisfactory. The CLS algorithm achieves high accuracy but with slightly degraded performance relative to the two-step estimator. The greatest advantage of the CLS estimator is its simplicity and relatively good performance. In addition, its fast convergence adds high potential for real-time tracking. The SPD estimator performs poorly when the ranging error is below −7 dB, but at high ranging error condition, it achieves better accuracy than the two-step estimator and the others. The LLS estimator’s MSE curve is almost a straight line, implying its accuracy being tightly dependent on the ranging error. In general, the LLS estimator’s performance is poor.

### Algorithm complexity

The complexity of many interior-point algorithms for solving SDP problems was studied in [43–45] and the references therein. One of the well-known results in the literature is that it takes *O*(*n*) iterations, where *n* denotes the size of the matrix variable involved, for the so-called large-step algorithm described in [45] to converge, where each iteration employs a Nesterov-Todd (N-T) search direction [43]. Since the complexity of computing an N-T direction in terms of number of multiplications is known to be in the order of *O*(*m* *n* ^3^+*m* ^2^ *n* ^2^) where *m* denotes the number of equality constraints involved [43], the complexity of the SDP algorithm in [45] is in the order of *O*(*m* *n* ^4^+*m* ^2^ *n* ^3^). In the context of the SDP problem in Eq. (14) where both the matrix size and the number of equality constraints are in the order of *M*, the complexity of solving problem (14) is in the order of *O*(*M* ^5^). Furthermore, the complexity of the second step in the algorithm in Section 4 is dominated by the computations required to compute the increment vector *Δ* **x** in Eq. (20), which is essentially equivalent to that of solving the positive definite linear system of equations

36 $$ \mathbf{(A^{T}W^{-1}A)\Delta \mathbf{x} = A^{T}Wb}.  $$

It is well known that the complexity of solving the above system of equations is about *M* ^3^/3. Since the algorithm needs to solve the SDP problem (14) only once plus K iterations in step two, the complexity of the algorithm in Section 4 is in the order of *O*(*M* ^5^)+*K* *M* ^3^/3.

For comparison, the complexity of $\hat {K}$ CLS iterations is in the order of $(\hat {K} M^{3})/3$ because the dominating computation required in each CLS iteration is to evaluate vector **H** ^−1^(**x**_*i*_)·∇*b*(**x** ^*k*^,*ϕ* ^*k*^) which can be done by solving the positive definite linear system of equations

37 $$ \mathbf{H}(\mathbf{x}_{i}) d_{k} = \nabla b(\mathbf{x}^{k},\phi^{k})  $$

for *d*_*k*_. We remark that above complexity analysis for the CLS method does not take the SVD of matrix **B** into account because **B** is a constant matrix (see Eq. (22b)) whose SVD can be performed off-line before the system starts to operate.

## Conclusions

An ATDOA positioning system and two associated location estimation algorithms are presented in this paper. The distinct advantage of the ATDOA system is that no clock synchronization is needed. Therefore, the complexity of the system can be reduced significantly. Besides, by properly selecting the anchor Rx node, the ATDOA system can achieve superior performance. In practice, as noise variance is dependent on the ranging distance, we have adopted a distance-dependent noise model to derive CRLB and to conduct simulations.

More importantly, two new localization algorithms, namely, the two-step and constrained LS algorithms have been proposed to provide position estimation in the ATDOA system. The two-step estimator combines the SDP and Taylor series methods to achieve global convergence and superior estimation accuracy. The constrained LS algorithm obtains good performance while keeps the computational complexity low, and the convergence speed is fast. Simulation results indicate that both estimators are able to achieve great performance regardless of the measurement error level. For the time-based localization systems, such as TOA, TDOA, ATDOA, and so on, the ranging error is relatively low, and under this condition, the two-step estimator achieves the best accuracy. In addition, its estimation accuracy is quite consistent regardless of the target node location and ranging error. Therefore, it can be applied in applications where accuracy is the most critical. The CLS estimator’s performance is slightly worse than the two-step estimator, nevertheless, it consumes less CPU time and requires lower computational complexity. Hence, it is very useful in real-time systems and mobile devices where battery life and computational capability is limited. In this regard, these two algorithms may be considered as a complementary pair of solution tools that provide the system designer with more than one option for an appropriate trade-off between accuracy and complexity.

## Appendix. proof of result (14)

By denoting *h*_*i*_=∥**x**−**x**_*i*_∥ and **h**=[*h*_1_,*h*_2_,⋯,*h*_*M*_]^*T*^, and dropping the terms in (13) that have no effects on the minimization, the ML cost function can be expressed as a constrained optimization problem,

38a $$\begin{array}{*{20}l} &\arg \mathop {\min }\limits_{\textbf{x},\textbf{h}} \sum\limits_{i = 2}^{M} {\frac{{{{{h}}_{i}}^{2} + 2 \cdot {{{h}}_{i}} \cdot {{{h}}_{1}} + {{{h}}_{1}}^{2}}}{{\sigma_{i}^{2}}}}  \\ & - \frac{{2 \cdot {{{h}}_{i}}\left({\left\| {{\textbf{x}_{i}} - {\textbf{x}_{1}}} \right\| + {r_{i}}} \right) + 2 \cdot {{{h}}_{1}}\left({\left\| {{\textbf{x}_{i}} - {\textbf{x}_{1}}} \right\| + {r_{i}}} \right)}}{{\sigma_{i}^{2}}}  \end{array} $$

38b $$\begin{array}{*{20}l}[-5pt] & \mbox {subject to:}  \\[-3pt] & h_{i} = \Arrowvert \mathbf{x} - \mathbf{x}_{i} \Arrowvert.  \end{array} $$

The cost function in (38a) remains non-linear because of the terms ${h}_{i}^{2}, {h}_{i}\cdot {h}_{1},$ and ${h}_{1}^{2}$. By introducing a parameter **H**=**h** **h** ^*T*^ and letting *h*_*i*_ *h*_1_=*h*_*i*1_, the problem at hand becomes

39a $$\begin{array}{*{20}l} &\arg \mathop {\min }\limits_{\textbf{x},\textbf{h},\textbf{R}} \sum\limits_{i = 2}^{M} {\frac{{{h_{ii}} + 2 \cdot {h_{i1}} + {h_{11}}}}{{\sigma_{i}^{2}}}}  \\ & - \frac{{2 \cdot {h_{i}}\left({\left\| {{\textbf{x}_{i}} - {\textbf{x}_{1}}} \right\| + {r_{i}}} \right) + 2 \cdot {h_{1}}\left({\left\| {{\textbf{x}_{i}} - {\textbf{x}_{1}}} \right\| + {r_{i}}} \right)}}{{\sigma_{i}^{2}}}  \end{array} $$

39b $$\begin{array}{*{20}l}[-8pt] & \mbox {subject to:}  \\[-3pt] & \mathbf{H} = \mathbf{hh}^{T}  \end{array} $$

39c $$\begin{array}{*{20}l} & h_{ii} = \mathbf{x}^{T}\mathbf{x} + \mathbf{x}_{i}^{T}\mathbf{x}_{i} - 2\mathbf{x}_{i}^{T}\mathbf{x}, i = 1, 2, \dots, M.  \end{array} $$

Furthermore, by introducing a new variable *z*=**x** ^*T*^ **x** to linearize constraint (39c), the above problem can be relaxed to a standard SDP problem which yields the result of (14).
